# Isoflurane lowers the cerebral metabolic rate of oxygen and prevents hypoxia during cortical spreading depolarization *in vitro*: An integrative experimental and modeling study

**DOI:** 10.1177/0271678X231222306

**Published:** 2023-12-23

**Authors:** Karl Schoknecht, Mathilde Maechler, Iwona Wallach, Jens P Dreier, Agustin Liotta, Nikolaus Berndt

**Affiliations:** 1 Carl-Ludwig-Institute of Physiology, Medical Faculty, Leipzig University, Leipzig, Germany; 2 Department of Anesthesiology and Intensive Care, Charité – Universitätsmedizin Berlin, Corporate Member of Freie Universität Berlin and Humboldt-Universität zu Berlin, Berlin, Germany; 3 Institute of Neurophysiology, Charité – Universitätsmedizin Berlin, Corporate Member of Freie Universität Berlin and Humboldt-Universität zu Berlin, Berlin, Germany; 4 Institute of Computer-Assisted Cardiovascular Medicine, Deutsches Herzzentrum der Charité (DHZC), Berlin, Germany; 5 Charité – Universitätsmedizin Berlin, Corporate Member of Freie Universität Berlin and Humboldt-Universität zu Berlin, Berlin, Germany; 6 Centre for Stroke Research Berlin, Charité – Universitätsmedizin Berlin, Corporate Member of Freie Universität Berlin and Humboldt-Universität zu Berlin, Berlin, Germany; 7 Department of Experimental Neurology, Charité – Universitätsmedizin Berlin, Corporate Member of Freie Universität Berlin and Humboldt-Universität zu Berlin, Berlin, Germany; 8 Department of Neurology, Charité – Universitätsmedizin Berlin, Corporate Member of Freie Universität Berlin and Humboldt-Universität zu Berlin, Berlin, Germany; 9 Bernstein Centre for Computational Neuroscience Berlin, Berlin, Germany; 10 Einstein Centre for Neurosciences Berlin, Berlin, Germany; 11 Institute of Health at Charité – Universitätsmedizin Berlin, Berlin; 12 Neuroscience Research Center, Charité – Universitätsmedizin Berlin, Corporate Member of Freie Universität Berlin and Humboldt-Universität zu Berlin, Berlin, Germany; 13 German Institute of Human Nutrition Potsdam-Rehbruecke (DIfE), Department of Molecular Toxicology, Nuthetal, Germany

**Keywords:** Cerebral metabolic rate of oxygen, spreading depolarization, isoflurane, hypoxia, cerebral oxygenation

## Abstract

Cortical spreading depolarization (SD) imposes a massive increase in energy demand and therefore evolves as a target for treatment following acute brain injuries. Anesthetics are empirically used to reduce energy metabolism in critical brain conditions, yet their effect on metabolism during SD remains largely unknown. We investigated oxidative metabolism during SD in brain slices from Wistar rats. Extracellular potassium ([K^+^]_o_), local field potential and partial tissue oxygen pressure (p_ti_O_2_) were measured simultaneously. The cerebral metabolic rate of oxygen (CMRO_2_) was calculated using a reaction-diffusion model. By that, we tested the effect of clinically relevant concentrations of isoflurane on CMRO_2_ during SD and modeled tissue oxygenation for different capillary pO_2_ values. During SD, CMRO_2_ increased 2.7-fold, resulting in transient hypoxia in the slice core. Isoflurane decreased CMRO_2_, reduced peak [K^+^]_o_, and prolonged [K^+^]_o_ clearance, which indicates reduced synaptic transmission and sodium-potassium ATPase inhibition. Modeling tissue oxygenation during SD illustrates the need for increased capillary pO_2_ levels to prevent hypoxia. In the absence thereof, isoflurane could improve tissue oxygenation by lowering CMRO_2_. Therefore, isoflurane is a promising candidate for pre-clinical studies on neuronal survival in conditions involving SD.

## Introduction

Cortical spreading depolarization (SD) was first described by Aristides Leão more than a half-century ago.^1^ Meanwhile, many experimental and clinical studies have detected SD in the course of critical brain disease, e.g. ischemic stroke, subarachnoid hemorrhage, spontaneous intracerebral hemorrhage, or traumatic brain injury (TBI).^2^ Importantly, SD has been associated with tissue deterioration, secondary brain injury, and reduced outcomes in patients.^3^ The duration of SD-induced spreading depression of cortical activity was the strongest predictor of delayed cerebral infarction in a recent clinical trial on subarachnoid hemorrhage.^4^ SD, therefore, evolves as a candidate biomarker for monitoring and treatment after brain insults.

SD is characterized by an extensive translocation of ions between the intracellular and extracellular space, which practically occurs together with membrane depolarization. Neuronal depolarization occurs slightly before astroglial depolarization.^5^ While Na^+^, Ca^2+^, and Cl^−^ ions enter neurons, K^+^ ions exit them. The K^+^ exit seems to occur slightly before the Na^+^, Ca^+^, and Cl^−^ entry in time.^6,7^ Overall, ion changes during SD are the largest that can occur in living neural tissue.^8^ As a consequence of neuronal depolarization, voltage-gated Na^+^ channels are inactivated, which prevents neuronal firing and causes the spreading depression of neuronal activity.^9–11^ However, the activity depression usually lasts significantly longer than the SD, suggesting that it is maintained by other mechanisms than the depolarization block, such as intracellular zinc or Ca^2+^ and/or extracellular adenosine accumulation.^12–14^ Morphologically, SD is associated with dendritic and somatic swelling, which is termed cytotoxic edema.^15^ To terminate SD, ion gradients have to be restored by increased activity of the sodium-potassium-ATPase (Na^+^/K^+^-ATPase).^16^ This, in addition to the need for increased tissue clearance of accumulated metabolites, is assumed to be one of the reasons why hyperemia occurs in otherwise healthy tissue during the depolarization phase in mammalian species, such as rats, pigs, cats, and humans, somewhat outlasting tissue repolarization and then followed by mild oligemia.^2^ However, despite hyperemia, tissue hypoxia may occur during SD even in otherwise intact tissues, because the increased cerebral metabolic rate of oxygen (CMRO_2_), which results from increased ATP demand, may outweigh oxygen delivery by increased regional cerebral blood flow (CBF).^17^ Only a few studies investigated oxygen consumption indicative of energy demand during SD. Those consistently showed elevations of CMRO_2_ in rats.^17–19^ In addition, brain ATP levels were shown to be reduced following KCl-induced SD in non-injured cortex in rats,^20^ indicating that ATP consumption exceeds production. Following brain insults, SD can aggravate brain damage.^3^ The situation becomes even more dangerous for the tissue when the neurovascular response to SD becomes inverse and instead of initial vasodilation and spreading hyperemia, severe vasoconstriction and spreading ischemia co-occur with simultaneously increased CMRO_2_.^21–25^ In the clinic, various conditions, such as subarachnoid hemorrhage, the penumbra in ischemic cerebral infarction, and TBI show a very similar continuum as in other mammals and especially in rats, ranging from brief hyperemic/hypoxic to prolonged ischemic/severe hypoxic responses to SD.^26–29^ In addition to normalizing the inverse hemodynamic response to SD in at-risk tissues, the reduction of SD-associated metabolic stress with a lower elevation of CMRO_2_ and corresponding lower hypoxia provides another conceivable and testable therapeutic option to reduce SD-induced secondary brain injury.

As anesthetics are known to reduce brain metabolism,^30–33^ they may reduce SD-associated damage that results from a mismatch of energy supply and demand. In addition, anesthetics may modulate or even prevent SD as shown for ketamine and isoflurane in animals and ketamine in patients.^34–39^ Empirically, deep anesthesia is used for neuroprotection in severe brain diseases, such as status epilepticus, TBI, stroke, and intracranial hypertension.^40,41^ However, the level of evidence for these indications of anesthesia is uncertain.^42^ Of note, deep anesthesia has been associated with poor outcomes after surgical treatment and neurocritical care,^43–45^ which implies that patients eligible to receive anesthesia for potential neuroprotection must be carefully selected. To choose the anesthetic with optimal protective properties and low neurotoxicity, it is of great interest to understand their effects on neuronal metabolism including the underlying molecular mechanisms. Anesthetics influence energy demand in the brain by suppressing neuronal activity,^33,46^ generating changes in regional CBF,^33,47^ and direct inhibition of mitochondrial enzymes.^46,48^ In particular, the gas anesthetic isoflurane has been shown to reduce cerebral metabolism by inhibiting synaptic transmission and network activity, e.g. by inhibiting N-methyl-D-aspartate receptors at the glycine site.^33,49^ In addition, transmitter release may be reduced due to direct inhibition of complex I of the respiratory chain in presynaptic terminals.^48^ Moreover, isoflurane was shown to impair the Na^+^/K^+^-ATPase,^50^ the major ATP consumer in the brain.^51^ Anesthetics that limit the great energy demand during SD and preserve metabolism may subsequently reduce hypoxia in acutely brain-injured patients.

In this study, we tested the effects of isoflurane on oxygen consumption during SD in neocortical slices of Wistar rats. We took advantage of the acute brain slice preparation to study neuronal energy demand under a constant supply of nutrients and oxygen. To quantify SD-associated metabolic demand and its modulation by isoflurane, we integrated recordings of the local field potential (LFP), extracellular potassium ([K^+^]_o_), and partial tissue oxygen pressure (p_ti_O_2_) during SD with *in silico* calculation of CMRO_2_. Using a tissue model, we extrapolated changes in CMRO_2_ during SD to oxygen availability *in vivo* for a range of previously reported capillary pO_2_ values.

## Material and methods

### Animals

This study was conducted in nine male and five female Wistar rats (Janvier Labs, weight: 250 g, age: ∼8 weeks) and complies with the ARRIVE 2.0 and the Charité Animal Welfare Guidelines. The experimental protocols were approved by the State Office of Health and Social Affairs of Berlin (T-CH0039/21). Before experiments, the animals had at least seven days for acclimation and were housed in groups of two with access to food *ad libitum* and a 12-h light/dark cycle.

### Slice preparation and maintenance

Animals were anesthetized using isoflurane/N_2_O (1.5%/70%, respectively) and decapitated. The brain was gently removed and coronal slices from the frontal cortex (thickness: 400 μm) were prepared with a Leica VT 1200 S vibratome (Wetzlar, Germany). Slices were immediately transferred to an interface chamber, where they were supplied with humidified carbogen (95% O_2_, 5% CO_2_, 1L/min, temperature ∼36 ± 0.5 °C) from the top and carbogenated artificial cerebrospinal fluid (aCSF) from the bottom at a flow of 2 mL/min.^52^ The aCSF contained (in mM): 129 NaCl, 26 NaHCO_3_, 10 glucose, 3 KCl, 1.25 NaH_2_PO_4_, 1.6 CaCl_2_, and 1.8 MgCl_2_ (osmolarity 295–305 mosmol/L, pH 7.35–7.45, temperature ∼36 ± 0.5 °C). Experiments started two hours after slicing.

### Electrophysiological and ptiO_2_ recordings and SD induction

**Figure 1.**
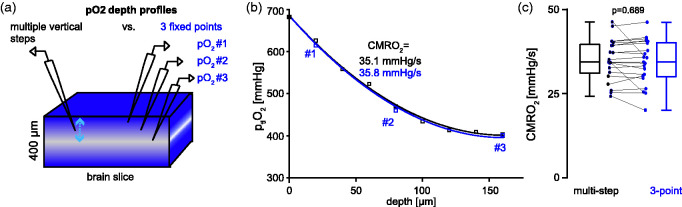
Three-point partial tissue oxygen pressure (p_ti_O_2_) recordings and cerebral metabolic rate of oxygen (CMRO_2_) calculations. a) Exemplary setup for measuring p_ti_O_2_ depth profiles using either one Clark-Style electrode for multiple consecutive vertical steps (left) from the slice surface to the core (blue arrow) in steps of 20 µm (left) or three electrodes at fixed positions from the surface to the core (right). Three fixed electrodes were used to increase temporal resolution, which allowed dynamic measurements during spreading depolarization (SD) (cf. Figure 2(b)). b) Exemplary baseline (pre-SD) p_ti_O_2_ depth profiles comparing multiple-vertical step recordings (black) with recordings by three fixed electrodes (blue). c) Bar graph summarizing baseline CMRO_2_ calculations based on multi-step and three-point p_ti_O_2_ recordings (n = 24 slices, ten animals, p = 0.69, Wilcoxon signed-rank test). Note the similarity of depth profiles (B) and subsequent CMRO_2_ calculations (C) for multi-step and three-point recordings.

Simultaneous LFP, [K^+^]_o_, and tissue p_ti_O_2_ measurements were performed in layer 2 using double-barrel ion-sensitive microelectrodes and Clark-type oxygen sensors (10 µm tip; Unisense, Aarhus, Denmark) as reported previously.^53^ Baseline CMRO_2_ was calculated based on p_ti_O_2_ measurements in vertical steps of 20 μm beginning at the slice surface until reaching the minimum of p_ti_O_2_ (i.e. core) as described previously.^54^ Stepwise measurements are time-consuming and therefore do not allow dynamic measurements during SD. To overcome this limitation, following baseline measurements, three oxygen probes were inserted at different depths (40 µm, 100 µm, and the measured core; Figure 1) in the same region. Ion-sensitive microelectrodes were manufactured using Potassium Ionophore I (Fluka, Buchs, Switzerland) as previously described.^55^ Recorded potentials were converted to [K^+^]_o_ in mM using Nernst’s equation and assuming baseline [K^+^]_o_ of 3 mM. Oxygen electrodes were polarized overnight and two-point calibrated before experiments. SDs were induced by local application of 3 M KCl at >200 μm from the recording sites with a glass micropipette before, during, and after the application of isoflurane. Data concerning LFP and [K^+^]_o_ recorded in five animals are published by Reiffurth *et al*., 2023.^50^ To exclude significant changes on SD properties and energy demand due to repeated SD induction with KCl, we performed control experiments without pharmacological treatment with isoflurane. For this purpose, four consecutive SDs were induced with repeated local application of 3 M KCl every 10–12 minutes (see Supplementary Fig. 1).

### Isoflurane application

Isoflurane was applied to an interface recording chamber with carbogen using a calibrated isoflurane vaporizer (Dräger, Germany) connected between the gas supply and the recordings chamber (constant gas flow rate of 1.5 l/min). The concentration of isoflurane was titrated to 1 and 3% vol. and controlled using a Vamos® mobile isoflurane monitor connected to a gas supply (Dräger, Germany). The recording temperature was maintained at ∼36°C. Given a water/gas partition coefficient of 0.5424 at 37 °C, the application of 1% and 3% correspond to 0.24 mM and 0.72 mM isoflurane in the aCSF, respectively.^56^

### Data acquisition and analysis

Analog signals were digitalized with Power CED1401 and Spike2 software (Cambridge Electronic Design, Cambridge, UK). Changes in ion concentrations were calculated using a modified Nernst equation as a mathematical expression for the generation of virtual channels on Spike2. Analyses and statistics were performed using Spike2, Excel (Microsoft, Seattle, USA), MATLAB (MathWorks Inc., Natick, MA, USA), and Origin (Version 6, Microcal Software, Northampton, USA).

### Calculation of CMRO_2_

CMRO_2_ was calculated from p_ti_O_2_ depth profiles as previously described.^52^ In short, we applied a reaction-diffusion model consisting of diffusive O_2_-transport and O_2_-consumption within the slice. Slices were divided into layers with an equal thickness of 1 μm. The diffusive oxygen distribution between the layers is described by Fick’s Law with a diffusion constant of 1.6 × 10^3^ μm^2^/s. The oxygen consumption rate within each layer is given by Michaelis-Menten kinetics (Km-value: 3 mmHg). The CMRO_2_ was assumed to be homogeneous throughout the slice and is treated as an adjustable parameter to match the experimental data. For the boundary conditions, the p_ti_O_2_ concentration at the slice surface was fixed to the supply value, while at the p_ti_O_2_ minimum, the diffusive oxygen transport was put to zero.

### Modeling perivascular oxygen diffusion

To simulate *in vivo* oxygen availability based on the calculated CMRO_2_ values during SD, we used a two-dimensional tissue model.^57,58^ The tissue that needs to be supplied is modeled as a cylinder constituted by a central capillary, which provides oxygen to the surrounding metabolically active neuronal tissue. Such a model was first described by A. Krogh.^59^ The radius of the Krogh cylinder is assumed to be in the range of 10–35 μm corresponding to reported radiuses of perivascular diffusion cylinders including the repeatedly reported average intercapillary distance of 40 μm (i.e., two Krogh cylinders with a radius of 20 μm).^60,61^ Oxygen diffuses from the vessel into the surrounding tissue where it is consumed. Oxygen diffusion is modeled by a compartmental discretization subdividing the cylinder in a vessel compartment and concentric tissue compartments around the vessel with a thickness of 1 µm. It is assumed that no oxygen leaves the cylinder, which is equivalent to the assumption that the outflow of oxygen from the represented region is equal to the inflow from neighboring regions.

### Statistical analysis

This was an exploratory study. We chose sample sizes that are common in the field and based on our own experience. Slices were subject to standardized wash-in protocols after recording SDs in standard conditions. This experimental design precluded the need to allocate slices to separate groups randomly and for anonymizing. No data were excluded.

Data were not normally distributed and are reported as median (25th, 75th percentile). Statistical inference was based on the Wilcoxon signed-rank test. P-values were adjusted for multiple comparisons by Bonferroni post hoc correction. Drug effects of 1% and 3% isoflurane were each compared with control but not with each other. Changes were stipulated to be significant for p-values <0.05.

## Results

### Three-point recordings allow measurements of p_ti_O_2_ depth profiles and CMRO_2_ with a high temporal resolution

**Figure 2.**
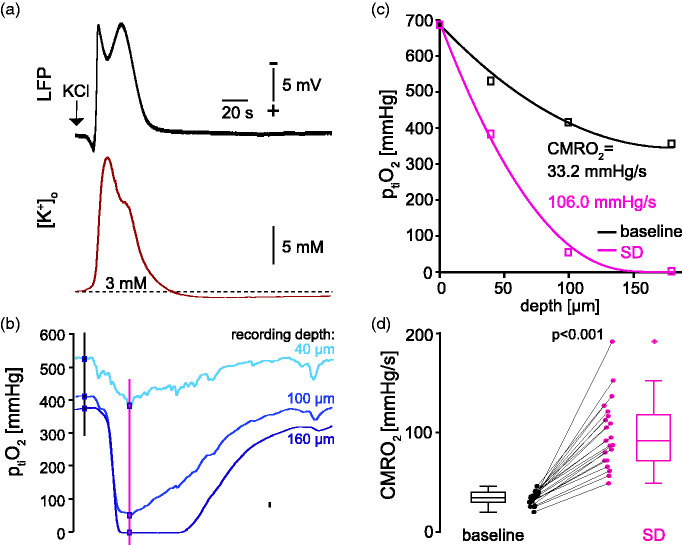
Exemplary time-dependent p_ti_O_2_ traces for multi-electrode recording during SD. a) Direct current (DC) local field potential (LFP) (top) and [K^+^]_o_ recording (bottom) of one SD induced by a KCl droplet. b) Parallel recording of p_ti_O_2_ at a depth of 40 (top), 100 (middle), and 160 µm (bottom) corresponding to the SD in (A). Vertical lines indicate time points of baseline and SD-associated depth profiles (black and magenta, respectively) used to calculate CMRO_2_ (cf. C). Note that the DC potential and the [K^+^]_o_ in A) return to baseline within ∼1 min while the p_ti_O_2_ is still reduced. c) Depth profiles taken before (black) and during SD (magenta) from the example shown in (B) and each corresponding CMRO_2_. d) Summary of CMRO_2_ values before (baseline) and during SD (n = 21 slices, ten rats, p < 0.0001, Wilcoxon signed-rank test).

Previously, calculations of CMRO_2_ relied on p_ti_O_2_ depth profiles acquired by vertical movement of one Clark-style oxygen-electrode in constant steps through acute brain slices (Figure 1(a) and (b)).^30,46,52,54^ This method can be used to capture steady-state CMRO_2_, but lacks the temporal resolution necessary for capturing the transient paroxysmal changes in p_ti_O_2_ during SD, which reach a minimum seconds after onset (cf. Figure 2(b)). To overcome this limitation, we established multielectrode recordings of p_ti_O_2_-depth profiles using three stationary electrodes inserted at fixed vertical positions in the slice (Figure 1(a)). We verified the accuracy of stationary three-electrode recordings with multi-step single electrode measurements (>7 vertical positions) at baseline before SD induction. As shown in Figure 1(b), depth profiles and CMRO_2_s were similar for single-electrode multi-step and stationary multi-electrode measurements (n = 21 slices, ten animals, p = 0.689, Wilcoxon signed-rank test), respectively (Figure 1(c)).

### SD causes a 2.7-fold increase in CMRO_2_

Next, we used the multi-electrode measurements to record rapid changes in the p_ti_O_2_ depth profiles and the related CMRO_2_ during SD. Focal application of KCl reliably initiated SD, which showed the characteristic negative deflection of the direct current (DC) potential and the increase in [K^+^]_o_ (Figure 2(a)). Exemplary traces for the three stationary oxygen electrodes demonstrate reduced p_ti_O_2_ in all depths. Of note, the return of p_ti_O_2_ to baseline levels after SD outlasted the recovery of the DC potential and of [K^+^]_o_. In the slice core, p_ti_O_2_ dropped below the hypoxia threshold of ∼8 mmHg at which oxidative metabolism breaks down (Figure 2(b)).^62^ Under a constant supply of oxygen, a drop of p_ti_O_2_ indicates increased oxygen consumption. Indeed, CMRO_2_ increased from 33.2 at baseline to 106.0 mmHg/s during SD in this example (Figure 2(c)) and ∼2.7-fold from 34.4 (30.8, 40.1) mmHg/s to 92.0 (72.0, 117.9) mmHg/s in summary (n = 21 slices, 10 animals, p < 0.0001, Wilcoxon signed-rank test, Figure 2(d)).

### Isoflurane reduces CMRO_2_, peak [K^+^]_o_, and prolongs SD and [K^+^]_o_ decay during SD

**Figure 3.**
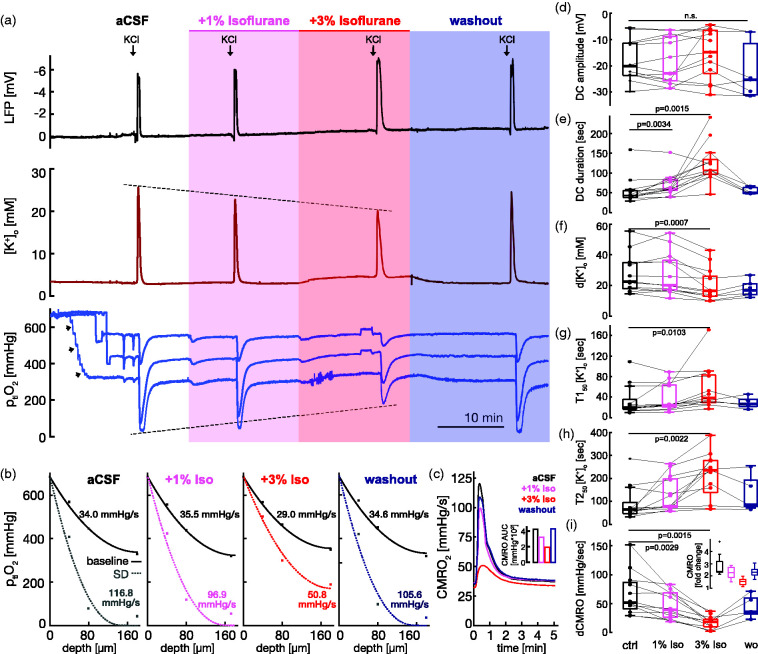
Effect of isoflurane on LFP, [K^+^]_o_, p_ti_O_2_, and CMRO_2_ during SD. a) Exemplary recordings of four KCl-induced SDs during perfusion of aCSF with the addition of 1% and 3% isoflurane, and during washout of isoflurane by aCSF. Arrows labeled with ‘KCl’ indicate the time point of KCl application by a glass microelectrode. Recorded were (from top to bottom) LFP, [K^+^]_o_, and p_ti_O_2_ at a depth of 40, 80, and 180 µm. Arrowheads in p_ti_O_2_ recording point to measurements of multi-step depth profiles at the beginning of the experiment. This electrode remains in the slice core thereafter. Note the successive lowering of peak [K^+^]_o_ and the increase in p_ti_O_2_ during SDs at 1% and 3% isoflurane. b) Depth profiles of p_ti_O_2_ before and during SD for the four conditions shown in (A), i.e. aCSF, aCSF + 1% isoflurane, aCSF + 3% isoflurane, and washout with aCSF. CMRO_2_s were calculated and displayed in the corresponding depth profiles. c) Dynamic display of CMRO_2_ for 5 min following SD onset corresponding to the example shown in A. Note the reduction of CMRO_2_ by 3% isoflurane at all time points relative to the onset of SD. The inset shows the cumulative CMRO_2_ calculated as the area under the curve (AUC) for the first 5 min following SD onset. Summary boxplots of d) SD-associated DC amplitudes, e) DC shift duration, f) Δ[K^+^]_o_, g) T1_50_ [K^+^]_o_, h) T2_50_ [K^+^]_o_, and i) CMRO_2._ The inset in I shows the change in CMRO_2_ during SD relative to the baseline before each SD.

We then investigated the influence of isoflurane on oxidative metabolism and [K^+^]_o_ dynamics during SD (Figure 3). We used concentrations clinically known to induce light and deep anesthesia, i.e., 1% and 3%. To avoid slice-dependent variability, a first SD was induced during perfusion of aCSF and served as a slice internal reference for subsequent SDs during the application of isoflurane and washout (Figure 3(a)). Neither 1% nor 3% isoflurane prevented the induction of SD and also had no significant effect on the SD-associated changes of the DC amplitude (Figure 3(a) and (d)). However, the duration of the SD-associated negative DC shift was prolonged from 43 sec (38, 55) to 60 sec (56, 83) and 107 sec (97, 134) for 1% and 3% isoflurane, respectively (Figure 3(e), n = 13 slices from nine rats, p = 0.0034 and p = 0.0015, respectively, Wilcoxon signed-rank test & Bonferroni correction). Furthermore, the example in Figure 3(a) shows a dose-dependent reduction of SD-associated [K^+^]_o_ peaks, a prolongation of the [K^+^]_o_-decay, and an increase of minimal p_ti_O_2_ levels above the hypoxia threshold. Corresponding depth profiles of p_ti_O_2_ and associated CMRO_2_ showed that isoflurane reversibly reduced oxygen consumption before and during SD (Figure 3(b)). We also calculated the CMRO_2_ continuously for 5 min following SD onset, which shows that 3% isoflurane reduced CMRO_2_ during the initial phase of SD, characterized by the negative shift of the DC potential and the rise in [K^+^]_o_, and also during the phase where [K^+^]_o_ returns to baseline levels (Figure 3(c)). The cumulative CMRO_2_ that exceeded baseline CMRO_2_ was approximately halved by 3% isoflurane during these 5 min. Before SD, CMRO_2_ was reduced from 38.9 mmHg/s (30.2, 41.0) during perfusion of aCSF to 34.7 mmHg/s (26.1, 36.8) following wash-in of 3% (but not 1%) isoflurane (p = 0.001, n = 12 slices, eight rats, Wilcoxon signed-rank test & Bonferroni correction). During SD, isoflurane significantly reduced the increase in CMRO_2_ (ΔCMRO_2_) from 51.8 (40.8, 86.6) mmHg/s during perfusion of aCSF to 39.4 (26.3, 68.3) and 18.4 (10.0, 22.7) mmHg/s at 1% and 3% isoflurane, respectively (p = 0.003 and p = 0.002, n = 12 slices, eight rats, Wilcoxon signed-rank test & Bonferroni correction, Figure 3(i)). In addition, isoflurane reduced the proportion of slices affected by core hypoxia from 58% (7 out of 12 slices) to 33% (4 out of 12 slices) and 17% (2 out of 12 slices) at 1% and 3% isoflurane, respectively. The peak change in [K^+^]_o_ (Δ[K^+^]_o_) during SD was reduced from 22.6 mM (17.8, 34.8) to 16.5 mM (13.0, 26.1) at 3% isoflurane (p = 0.001, n = 13 slices, nine rats, Wilcoxon signed-rank test & Bonferroni correction, Figure 3(f)), whereas 1% isoflurane had no significant effect. In addition to Δ[K^+^]_o_, we analyzed the [K^+^]_o_ decay time to 50% (T1_50_ [K^+^]_o_) and 10% (T2_50_ [K^+^]_o_) of the peak Δ[K^+^]_o_ during SD. 3% isoflurane increased both T1_50_ and T2_50_ from 20.0 sec (16.2, 35.3) to 38.3 sec (29.1, 82.9) and from 65.0 sec (47.7, 95.3) to 235.7 sec (139.5, 277.5), respectively (Figure 3(f) and (g), p = 0.01 and p = 0.002, respectively, n = 13 slices, nine rats, Wilcoxon signed-rank test & Bonferroni correction), whereas 1% isoflurane did not significantly alter [K^+^]_o_ decay times. Despite some slice- and sex-dependent heterogeneity, the effects of isoflurane were similarly observed in most slices and in males and females (Figure 3(d) to (i), Supplementary Table 1). Importantly, the effects of isoflurane on CMRO_2_, DC shift duration, Δ[K^+^]_o_, T1_50_ [K^+^]_o_, and T2_50_ [K^+^]_o_ were transient as washout of isoflurane partly restored all parameters (Figure 2(a) to (c), (e) to (h)). Furthermore, in control experiments in the absence of isoflurane, all measured parameters were stable during four consecutive SDs (n = 8 slices, three rats, Supplementary Fig. 1). In summary, isoflurane significantly prolonged SD duration and [K^+^]_o_ recovery, whereas Δ[K^+^]_o_ and CMRO_2_ were reduced.

### Isoflurane improves tissue oxygenation

**Figure 4.**
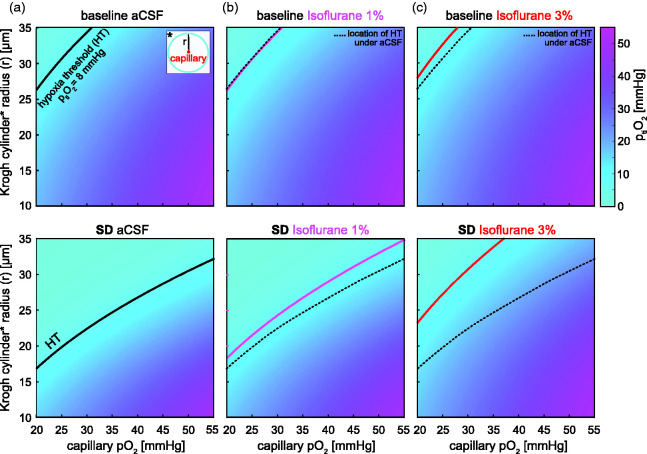
Modeling of oxygen diffusion based on CMRO_2_ during SD. a) Modeling of minimal p_ti_O_2_ levels depending on the radius (r) of Krogh cylinders (y-axis and inset) and the capillary pO_2_ (x-axis). The top and bottom panels show p_ti_O_2_ for the average CMRO_2_ measured before and during an SD, respectively, under the perfusion of aCSF. The black line indicates capillary pO_2_-dependent radiuses of Krogh cylinders where the hypoxia threshold (HT, ∼8 mmHg) would be reached. Note the considerable shift to smaller Krogh cylinders during SD. b) Panels equivalent to A for baseline (top) and SD-associated CMRO_2_ (bottom) under 1% isoflurane. Note that isoflurane increased the range of Krogh cylinders above HT during SD but not for baseline CMRO_2._ c) Panels equivalent to A for baseline (top) and SD-associated CMRO_2_ (bottom) under 3% isoflurane. Note that isoflurane increased the range of Krogh cylinders above HT before and during SD. The dotted black line (b,c) indicates the location of the HT in isoflurane-free aCSF as shown in a.

Given the 2.7-fold increase of CMRO_2_ during SD *in vitro*, we investigated how this would affect tissue oxygenation in a tissue model. Based on pO_2_ in the range of 20–55 mmHg, which has been recorded in capillaries of anesthetized and awake rodents,^60,63,64^ we simulated minimal p_ti_O_2_ within perivascular Krogh cylinders, i.e. at the edge of the cylinder, with radiuses in the range of 10 to 35 µm (see Figure 4(a)).^58,60,61^ For a capillary pO_2_ as low as 20 mmHg, we found oxygen supply >8 mmHg for Krogh cylinders with radiuses of up to 27 µm under baseline CMRO_2_ (see the top panel in Figure 4(a)). This extends beyond the midline of the reported average cortical intercapillary distance of ∼40 µm^58,60,61^ and thus shows sufficient oxygen supply to most brain tissue. However, the increased CMRO_2_ during SD induced a severe drop in p_ti_O_2_ thereby locally shifting the hypoxia boundary (p_ti_O_2_<8 mmHg, black line in Figure 4(a)) into the reported size range of Krogh cylinders.^60^ This highlights the need for an increase in capillary pO_2_ during SD to prevent hypoxia in some, although certainly not all brain tissue. To evaluate the effect of isoflurane, we repeated the simulation using the CMRO_2_s determined for 1% and 3% isoflurane. 1% isoflurane increased the radius of Krogh cylinders expected to receive >8 mmHg O_2_ during but not before SD, whereas 3% improved both baseline and SD-associated tissue oxygenation (Figure 4(b) and (c)).

## Discussion

Tissue oxygenation reflects the balance of oxygen supply and CMRO_2_. Monitoring tissue oxygenation with parenchymal probes has become a standard procedure after subarachnoid hemorrhage and TBI, i.e. conditions during which SD has been recorded.^3,26,27,65,66^ However, CMRO_2_ during SD has been studied only sparsely, although it is known that the associated energy demand increases dramatically.^3^ We, therefore, performed quantitative measurements of CMRO_2_ during SD in acute brain slices. *In vivo*, SD was shown to cause temporary hypoxia despite vasodilation in some cases.^17^ Therefore, lowering CMRO_2_ could improve tissue oxygenation and prevent hypoxia. Importantly, we have previously shown that isoflurane lowers cerebral metabolism, while neurovascular coupling to burst activity and mitochondrial respiration were preserved^33^. This made isoflurane a promising candidate for testing its lowering effect on CMRO_2_ during SD.

### CMRO_2_ increases 2.7-fold during SD

We investigated CMRO_2_ during SD in acute brain slices, which allows a constant supply of oxygen. Therefore, changes in p_ti_O_2_ depth profiles directly reveal changes in CMRO_2_. Using novel three-point recordings, we were able to continuously monitor p_ti_O_2_ depth profiles and thereby provide calculations of peak CMRO_2_ during SD *in vitro*. We found that CMRO_2_ increased ∼2.7 fold during SD. Quantitatively, this increase in CMRO_2_ was higher than in anesthetized rats (1.5–1.7-fold change) *in vivo*.^17–19^ CMRO_2_ calculations *in vivo* were derived from CBF measurements combined with p_ti_O_2_ recordings^17,19^ or combined with arterial-venous pO_2_ differences.^18^ The quantitative difference between these *in vivo* and our *in vitro* measurements, may have several reasons: (a) Supply of surplus oxygen via carbogen *in vitro* may allow greater CMRO_2_ than *in vivo* where provided oxygen may be fully consumed at lower CMRO_2_. (b) Neuronal activity before SD may be lower *in vitro*, which would augment the relative change in CMRO_2_ during SD in our experiments; (c) The absence of confounding effects of CBF may allow more accurate assessment of CMRO_2_ *in vitro*; and (d) due to the high temporal resolution of our recordings, we may have captured greater peak CMRO_2_.

Compared to our other *in vitro* studies, the SD-related increase in CMRO_2_ (2.7-fold) was significantly higher than during other energy-demanding types of network activity, such as gamma oscillations or seizure-like events, which induced a 1.3-fold and 1.4-fold increase in CMRO_2_, respectively.^30,54^ This underlines the great metabolic demand induced by SD.

### Isoflurane decreases CMRO_2_ during SD: possible mechanisms in light of [K^+^]_o_ dynamics

Isoflurane was shown to reduce CMRO_2_ in animals at concentrations that induced burst suppression,^33,67,68^ and in 12 patients suffering from subarachnoid hemorrhage.^69^ We show that isoflurane similarly reduced CMRO_2_ during SD by approximately 5% and 35% at concentrations of 1% and 3%, respectively (Figure 3(g), inset). These concentrations of isoflurane were chosen because they clinically correspond to light (1%) and deep (3%) anesthesia.

Isoflurane has multiple molecular targets that could reduce CMRO_2_ during SD, and [K^+^]_o_ recordings provide mechanistic hints. Isoflurane lowered peak [K^+^]_o_ thereby limiting the demand for oxidative metabolism to restore the transmembrane K^+^ gradient after SD. The mechanisms underlying the initial neuronal release of K^+^ have not been resolved. Subsequently, it is not known how isoflurane lowers peak [K^+^]_o_. Reduced synaptic transmission under isoflurane may explain lower peak [K^+^]_o_ values in the early phase of SD, when typically [K^+^]_o_ and the extracellular concentration of glutamate rise rapidly (see Figures 2 and 3 and Menyhart et al., 2022).^70^ Application of glutamate has been shown to elevate [K^+^]_o_.^71^ In parallel with the initial glutamate release and increase in [K^+^]_o_, the frequency of excitatory postsynaptic potentials increases during SD,^72^ although inactivation of voltage-gated Na^+^ channels causes depression of activity at the network level. Synaptic transmission could be reduced by isoflurane by inhibition of NMDA receptors,^49^ increased GABAergic input,^73^ opening of 2-pore-domain potassium channels,^74^ and impaired presynaptic Ca^2+^ influx.^75^ Furthermore, inhibition of mitochondrial complex I in presynaptic terminals by isoflurane has been shown to impair transmitter release.^48^ Together, these effects could reduce initial synaptic transmission during SD and thereby lower activity-dependent energy demand.

In contrast to the lowering of peak [K^+^]_o_, prolonged [K^+^]_o_ clearance by isoflurane cannot be explained by synaptic effects. However, this might result from reduced Na^+^/K^+^-ATPase activity.^76^ Conversely, the reduction in peak [K^+^]_o_ during SD was shown to be insensitive to pharmacological inhibition of the Na^+^/K^+^-ATPase^76^ and therefore cannot be explained by its reduced activity. Of note, we recently found a dose-dependent impairment of the Na^+^/K^+^-ATPase containing α2/3 subunits in the cortex of rat brains by isoflurane.^50^ Similar to [K^+^]_o_ clearance, the return of [Na^+^]_o_ to baseline levels following SD was prolonged in those experiments, which is in line with the inhibition of the Na^+^/K^+^-ATPase.^50^ Since the Na^+^/K^+^-ATPase is the greatest energy consumer in the brain,^51^ a decrease in its activity lowers oxygen consumption. Still, inhibition of the Na^+^/K^+^-ATPase may cause adverse events. The prolonged reduction of [Na^+^]_o_ following SD in the presence of isoflurane may have secondary effects on Ca^2+^ homeostasis, i.e. it may prolong the cellular Ca^2+^ overload, which could promote death signaling.^77^ One of the main transporters for the efflux of intracellular Ca^2+^ is the Na^+^/Ca^2+^ exchanger (NCX), which exchanges three extracellular Na^+^ ions for one intracellular Ca^2+^ ion.^78^ Elevated [Na^+^]_i_, as indicated by reduced [Na^+^]_o_, inhibits the Ca^2+^ exit mode of the NCX. Therefore, prolonged elevation of [Na^+^]_i_, as during Na^+^/K^+^-ATPase inhibition by isoflurane, may extend the intracellular Ca^2+^ surge during SD. On the other hand, ATP has been shown to increase the affinity of the NCX for intracellular Ca^2+^ and extracellular Na^+^ by enabling the phosphorylation of the transporter.^78^ Isoflurane may thus facilitate Ca^2+^ efflux via the NCX if it indeed prevents ATP shortages. Similarly, isoflurane may increase Ca^2+^-ATPase activity, although this is thought to contribute less to Ca^2+^ efflux than NCX due to a lower turnover rate.^79^ Isoflurane may also lower the SD-induced Ca^2+^ load itself because the dendritic Ca^2+^ influx during SD was shown to depend on NMDA receptor activation,^72^ which isoflurane inhibits.^49^ In addition, experiments on mouse brain slices suggest that the magnitude and mechanisms of the neuronal Ca^2+^ increase differ for SDs induced by hypoxia or by high [K^+^]_o_ under normoxia.^80^ Therefore, the effect of isoflurane on neuronal Ca^2+^ dynamics may also depend on the trigger of SD. In summary, while we provide evidence that isoflurane lowered CMRO_2_ during SD by reducing synaptic transmission as well as Na^+^/K^+^-ATPase activity, the neuronal outcome has yet to be investigated.

Several studies on cellular and functional outcomes following experimental ischemia suggest the protective effects of isoflurane. Isoflurane was shown to be neuroprotective in postnatal day 10 pups that underwent unilateral carotid ligation.^81^ Isoflurane also reduced cell damage in acute rat brain slices that underwent a period of oxygen-glucose deprivation.^82^ Following middle cerebral artery occlusion, isoflurane reduced infarct volume and improved neurological outcome 24 hours^82^ and four weeks after stroke.^83,84^ The suggested mechanisms that underlie neuroprotection include the sphingosine-1-phosphate/phosphatidylinositol-3-kinase/Akt pathway, activation of nuclear factor-κB, production of interleukin-1β, and increased expression of B-cell lymphoma-2 (Bcl-2) protein, all of which putatively result from hypoxia. Therefore, the reduction of secondary hypoxia during SD or prevention of SD by isoflurane, as shown for KCl-induced SD in non-ischemic tissue,^39^ may be a missing link to neuroprotection in these studies. In this context, it is of interest that isoflurane was shown to increase CBF in anesthetized rabbits and humans,^85,86^ presumably due to vasodilation, which in turn may improve tissue oxygenation and thus add to the putative protective effects secondary to reduced CMRO_2_. Of note, we did not demonstrate complete reversibility of isoflurane effects in all experiments. This could limit applicability, e.g. due to side effects of prolonged Na^+^/K^+^-ATPase inhibition (see previous paragraph).

### Translational relevance of reduced CMRO_2_ during SD

We next asked to what extent a reduction of CMRO_2_ during SD could improve tissue oxygenation. The tissue model predicted that SD will lead to a transient episode of hypoxia in some brain tissue in the absence of increased oxygen supply, e.g. by a physiological hyperemic blood flow response, which is typical in otherwise healthy tissue^2^. In conjunction with the experimental data, the tissue model generates and supports the hypothesis that it is feasible to lower CMRO_2_ during SD to a degree that would prevent critically low p_ti_O_2_ levels within the range of reported intercapillary distances.^60,61^ Therefore, the application of isoflurane could be beneficial in conditions when resting state CBF is maintained but the capacity to increase the supply of oxygen and energy-rich substrates by neurovascular coupling is impaired. This could affect pathological conditions such as subarachnoid hemorrhage, TBI, or the ischemic penumbra.^26–29,87^

In conclusion, clinically relevant concentrations of isoflurane could improve tissue oxygenation after SD by lowering the demand for oxidative metabolism, especially when the neurovascular coupling is impaired. Of note, the inhibitory effects of isoflurane on Na^+^/K^+^-ATPase activity could weaken or outweigh the protective effects of improved tissue oxygenation. Previously demonstrated neuroprotective effects of isoflurane following ischemia are a promising observation. Based on our data, we propose further studies focusing on metabolic effects during SD.

### Study limitations

For the calculation of CMRO_2_, we assumed the oxygen demand and the effective affinity of the respiratory chain enzymes to oxygen to be constant throughout individual slices. This is certainly an abstraction. Local variations in oxygen consumption are likely present due to the spatial arrangements of different cell parts, such as dendrites, soma, and axons due to different cell types, such as neurons, astrocytes, or microglia, and due to different local activity states. The inhomogeneous distribution of metabolic activity might also result from inhomogeneities in substrate supply (not only of oxygen but also of glucose and lactate). Furthermore, the slicing procedure induces tissue damage and disrupts the neuronal network thereby influencing local metabolic activity. However, although we are not able to dissect the sources of metabolic inhomogeneity, the assumption of homogenous oxygen consumption overall fits the measured oxygen depth profiles.

In addition, due to fundamental experimental differences between brain slice and intravital recordings (e.g. absence of CBF, constant surplus oxygen supply, distortion of neuronal networks), peak CMRO_2_ values during SD found *in vitro* may not reflect *in vivo* oxygen consumption accurately.

## Supplemental Material

sj-pdf-1-jcb-10.1177_0271678X231222306 - Supplemental material for Isoflurane lowers the cerebral metabolic rate of oxygen and prevents hypoxia during cortical spreading depolarization *in vitro*: An integrative experimental and modeling study Supplemental material, sj-pdf-1-jcb-10.1177_0271678X231222306 for Isoflurane lowers the cerebral metabolic rate of oxygen and prevents hypoxia during cortical spreading depolarization *in vitro*: An integrative experimental and modeling study by Karl Schoknecht, Mathilde Maechler, Iwona Wallach, Jens P Dreier, Agustin Liotta and Nikolaus Berndt in Journal of Cerebral Blood Flow & Metabolism

sj-pdf-2-jcb-10.1177_0271678X231222306 - Supplemental material for Isoflurane lowers the cerebral metabolic rate of oxygen and prevents hypoxia during cortical spreading depolarization *in vitro*: An integrative experimental and modeling study Supplemental material, sj-pdf-2-jcb-10.1177_0271678X231222306 for Isoflurane lowers the cerebral metabolic rate of oxygen and prevents hypoxia during cortical spreading depolarization *in vitro*: An integrative experimental and modeling study by Karl Schoknecht, Mathilde Maechler, Iwona Wallach, Jens P Dreier, Agustin Liotta and Nikolaus Berndt in Journal of Cerebral Blood Flow & Metabolism
