# Supplementary material for: Isoflurane lowers the cerebral metabolic rate of oxygen and prevents hypoxia during cortical spreading depolarization in vitro: An integrative experimental and modeling study
Source: J Cereb Blood Flow Metab. 2023 Dec 23;44(6):1000–12. doi: 10.1177/0271678X231222306 (PMC11318408; doi:10.1177/0271678X231222306)
Supplement: sj-pdf-1-jcb-10.1177_0271678X231222306 - Supplemental material for Isoflurane lowers the cerebral metabolic rate of oxygen and prevents hypoxia during cortical spreading depolarization in vitro: An integrative experimental and modeling study [file sj-pdf-1-jcb-10.1177_0271678X231222306.pdf]

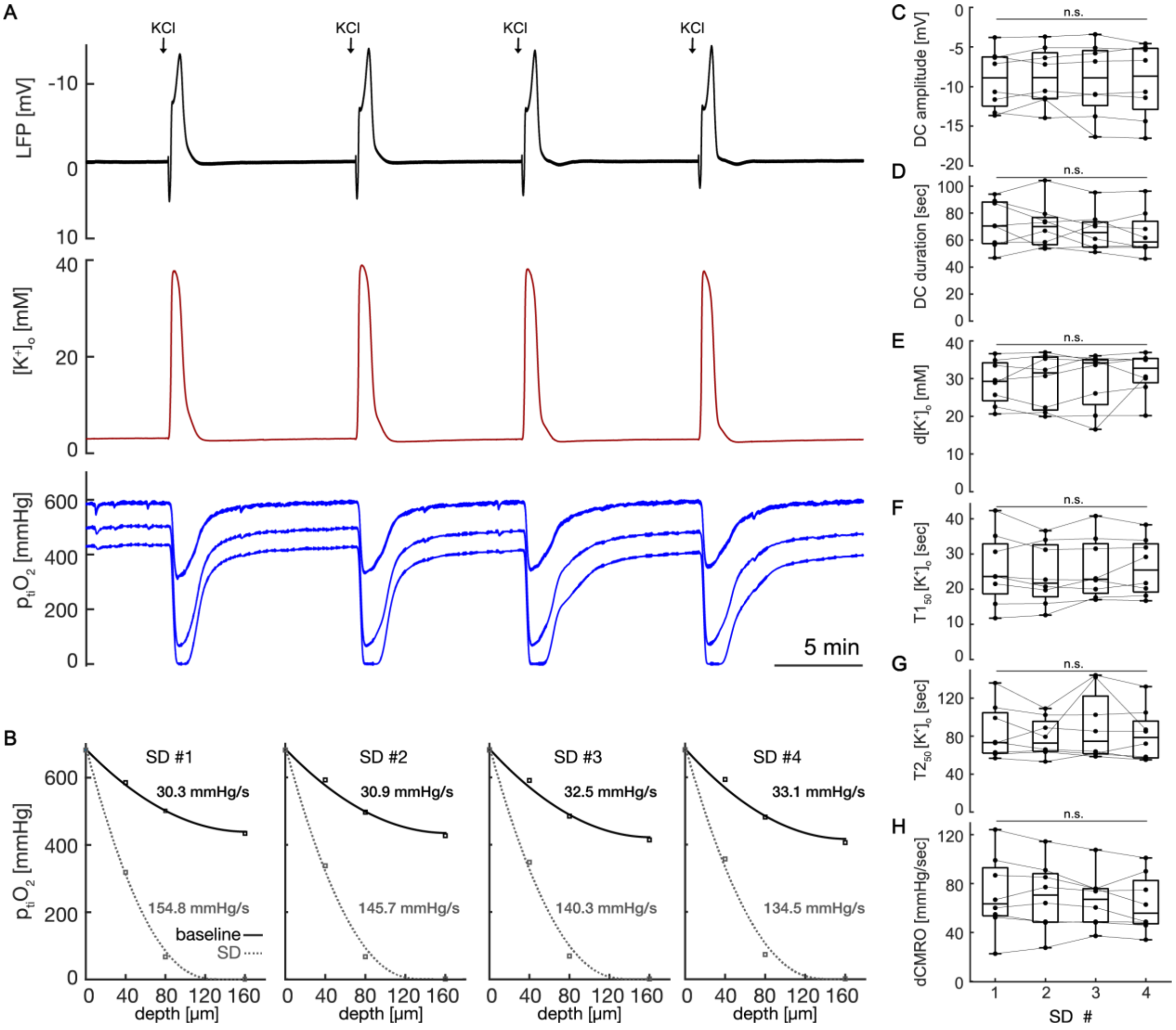

Supplementary Fig. 1 Recurrent spreading depolarizations (SDs) without application of isoflurane

Exemplary recordings of four consecutive KCl-induced SDs during perfusion of artificial cerebrospinal fluid (aCSF). Arrows labeled with 'KCl' indicate the time point of KCl application by a glass microelectrode. **A)** Recorded were (from top to bottom): local field potential (LFP), extracellular potassium $[K^+]_o$, and partial tissue oxygen pressure ($p_{ti}O_2$) at a depth of 40, 80, and 160 µm. **B)** Depth profiles of $p_{ti}O_2$ before and during the four SDs shown in (A). $CMRO_2$s were calculated and displayed in the corresponding depth profiles. Summary boxplots of **C)** SD-associated direct current (DC) amplitudes, **D)** DC shift duration, **E)** $\Delta[K^+]_o$, **F)** $T1_{50}$ $[K^+]_o$, **G)** $T2_{50}$ $[K^+]_o$, and **H)** $CMRO_2$ (n = 8 slices from three rats for all parameters, n.s. - not significant). Note the similarity of the four SDs in the exemplary recording and in the quantitative analysis.
