# Supplementary material for: Isoflurane lowers the cerebral metabolic rate of oxygen and prevents hypoxia during cortical spreading depolarization in vitro: An integrative experimental and modeling study
Source: J Cereb Blood Flow Metab. 2023 Dec 23;44(6):1000–12. doi: 10.1177/0271678X231222306 (PMC11318408; doi:10.1177/0271678X231222306)
Supplement: sj-pdf-2-jcb-10.1177_0271678X231222306 - Supplemental material for Isoflurane lowers the cerebral metabolic rate of oxygen and prevents hypoxia during cortical spreading depolarization in vitro: An integrative experimental and modeling study [file sj-pdf-2-jcb-10.1177_0271678X231222306.pdf]

Schoknecht *et al.*

Isoflurane lowers the cerebral metabolic rate of oxygen and prevents hypoxia during cortical spreading depolarization *in vitro*: an integrative experimental and modeling study

Supplementary Table 1 Sex-dependent summary of results

From top to bottom: Spreading depolarization-associated direct current (DC) amplitudes, DC shift duration, $\Delta[K^+]_o$ (extracellular potassium), $T1_{50}$ $[K^+]_o$, $T2_{50}$ $[K^+]_o$, and cerebral metabolic rate of oxygen ($CMRO_2$). Ctrl = control; Iso = isoflurane

**Male**	**DC amplitude [mV]**			**Female**			
n=7 slices, 4 rats	ctrl	Iso 1%	Iso 3%	n=6 slices, 5 rats	ctrl	Iso 1%	Iso 3%
**median**	-23.3	-25.4	-21.5		-15.8	-14.0	-12.6
25th percentile	-24.2	-27.2	-27.3		-19.9	-19.8	-15.6
75th percentile	-18.2	-20.6	-6.6		-11.8	-10.2	-9.2
n=7 slices, 4 rats	**DC shift duration [sec]**			n=6 slices, 5 rats			
**median**	41.9	58.5	106.9		56.4	80.9	125.5
25th percentile	38.7	56.5	98.2		46.6	67.7	110.8
75th percentile	43.6	61.1	118.3		70.1	86.7	128.2
n=7 slices, 4 rats	**$\Delta[K^+]_o$ [mM]**			n=6 slices, 5 rats			
**median**	18.8	17.7	14.0		34.1	37.3	27.2
25th percentile	15.8	17.2	13.0		29.9	30.6	24.8
75th percentile	22.0	19.7	15.8		40.0	43.8	33.2
n=7 slices, 4 rats	**$T1_{50}$ $[K^+]_o$ [sec]**			n=6 slices, 5 rats			
**median**	17.0	21.8	33.1		44.1	64.7	84.8
25th percentile	13.8	16.2	29.0		25.7	44.2	57.3
75th percentile	19.0	24.5	40.2		65.3	72.2	89.8
n=7 slices, 4 rats	**$T2_{50}$ $[K^+]_o$ [sec]**			n=6 slices, 5 rats			
**median**	48.2	79.4	239.7		117.5	164.0	193.0
25th percentile	46.1	74.7	221.0		70.0	105.0	120.0
75th percentile	56.1	91.2	277.5		166.0	202.0	237.5
n=6 slices, 3 rats	**$\Delta CMRO_2$ [mmHg/sec]**			n=6 slices, 5 rats			
**median**	40.8	37.6	18.8		72.3	52.9	15.3
25th percentile	33.3	28.5	10.9		51.8	33.6	11.1
75th percentile	57.1	51.3	22.7		101.3	72.0	19.0
